# Disinfection of *Escherichia coli* in ice by surface dielectric barrier discharge plasma

**DOI:** 10.1063/5.0064020

**Published:** 2021-08-30

**Authors:** Yuntao Guo, Peipei Liu, Liyang Zhang, Siqi Peng, Xinxin Wang, Haiyun Luo, Guizhen Wu

**Affiliations:** 1 Department of Electrical Engineering, Tsinghua University, Beijing 100084, China; 2 NHC Key Laboratory of Biosafety, National Institute for Viral Disease Control and Prevention, Chinese Center for Disease Control and Prevention, Beijing 102206, China

## Abstract

A variety of pathogens can cause people to suffer from serious diseases, and the transmission of COVID-19 through the cold chain has once again attracted people's attention to cold chain disinfection. Unfortunately, there is no mature cold chain disinfection technique yet. In this study, a low-temperature plasma disinfection technique for a cold chain is proposed. The disinfection effect of plasma generated by surface dielectric barrier discharge on *Escherichia coli* in ice at cryogenic temperature is studied, and the possible disinfection mechanism is discussed. It is found that the O_3_ mode and the NO_x_ mode also exist in the surface dielectric barrier discharge at cryogenic temperature, just as at room temperature. The disinfection effect of both modes is weak in 5 min plasma treatment, but in 60 min post-treatment, the NO_x_ mode shows a stronger disinfection effect, with 4.45 log reduction. It is speculated that gaseous H_2_O_2_ and NO_x_ can be adsorbed on the ice surface in the NO_x_ mode and then converted into peroxynitrite, which is a powerful bactericidal species. In conclusion, a low-temperature plasma is a promising technique for cold chain disinfection, which is of great significance for ensuring people's health.

*Escherichia coli*,^1^*Staphylococcus aureus*,^2^ human norovirus,^3^ and other foodborne pathogens^4,5^ can cause people to suffer from serious diseases. A cold chain plays an important role in food transportation and preservation, but concerns about food safety are often ignored.^6^ In the recent outbreak of COVID-19, Liu *et al.*^7^ reported that the severe acute respiratory syndrome coronavirus 2 (SARS-CoV-2) was directly isolated from the package surface of frozen cod, which was considered the source of infection in dock workers. Pang *et al.*^8^ speculated that the COVID-19 resurgence in Beijing was likely to be initiated by an environment-to-human transmission originated from contaminated imported food via cold-chain logistics. All the above cases are warning us that the contaminated foods in the cold chain could serve as a long-range carrier of pathogens, presenting a potential risk of its transmission across the regions and countries via cold chain industries.^9^ There is an urgent demand for disinfection in the cold chain to ensure the safety of the foods and the people involved.

Conventional thermal treatments can inactivate foodborne pathogens,^10^ but it obviously cannot be used for cold chain disinfection. In recent years, some non-thermal disinfection techniques have been developed, such as high-pressure treatment (HPP), ultraviolet (UV), ultrasonic, pulsed electric field (PEF), irradiation, and non-thermal plasma.^4,11^ However, there are few reports on disinfection techniques directly used in the cold chain. Baker *et al.*^12^ reported a peroxygen based disinfectant in a 10% propylene glycol solution cloud inactivate epidemic diarrhea virus on aluminum surfaces at −10 °C. Zhang^6^ proposed the concept of using liquid CO_2_ as a green and safe low-temperature disinfectant in the cold chain, but the disinfection effect was not verified by experiments.

Low temperature plasma, or named non-thermal plasma, is a novel disinfection technique with high efficiency and small side effects. Its disinfection effect has been widely studied, which can inactivate various foodborne pathogens such as bacteria,^13,14^ fungi,^15^ virus,^16^ and even SARS-CoV-2.^17–19^ Zhang *et al.*^20^ recently reported that the low-temperature plasma inactivated pseudoviruses with the SARS-CoV-2 S protein on the surface in a cold chain environment, but the study on the inactivation of pathogens in ice by the low-temperature plasma has not been reported. Due to the fact that the discharge process is almost independent of the ambient temperature, it is possible to generate a plasma by discharging at cryogenic temperature,^21^ which makes this technique has unique advantages in cold chain disinfection. In this paper, the disinfection effect of the low temperature plasma on *Escherichia coli* (*E. coli*, ATCC 8099) in ice is studied, and the possible disinfection mechanism is discussed.

The experimental setup is shown in Fig. 1. Surface dielectric barrier discharge (SDBD) was used to generate the low temperature plasma in this study, which was similar to that of other researchers with slight changes.^22,23^ Briefly, a round aluminum plate (40 mm diameter), a stainless-steel mesh (40 mm diameter, 60 mesh/cm^2^), and 1 mm ceramic plate were used as high voltage electrodes, ground electrodes, and dielectric barriers, respectively. A power source was used to supply a sinusoidal high voltage with the frequency of 1 kHz and the voltage amplitude in the range of 0–15 kV. The voltage and current were measured with a high voltage probe (Tektronix, P6015A) and a current probe (Tektronix, TCP312A), respectively. Another probe (Tektronix, TPP0200) was used to measure the voltage across a capacitor (65 nF) connected in series to the ground electrode. All waveforms were recorded using an oscilloscope (Tektronix, MDO3034). The power consumption was obtained by the Lissajous method^24,25^ and then the power density was calculated by dividing the power consumption by the electrode area.

**FIG. 1.**
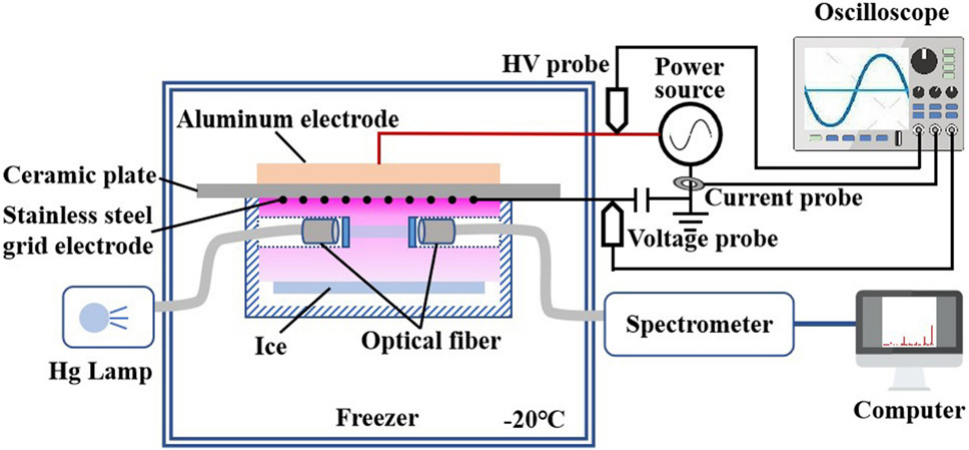
Schematic diagram of the experimental setup.

The power densities of SDBD at room temperature (30 °C) and cryogenic temperature (−20 °C) were measured, and the results are shown in Fig. 2(a). They are almost the same at the same voltage, and the difference is less than 7%. Taking 8 kV as an example, the shape and area of Lissajous figures [Fig. 2(d)] measured under these two temperatures are similar. The calculated power densities are 0.094 and 0.092 W/cm^2^, corresponding to −20 and 30 °C, respectively. When the voltage amplitude is 15 kV, the power densities are 0.327 and 0.319 W/cm^2^, respectively. As pointed out in the literature,^24,26^ the discharge area changes continuously during the SDBD process, resulting in the almond-shaped Lissajous figures.

**FIG. 2.**
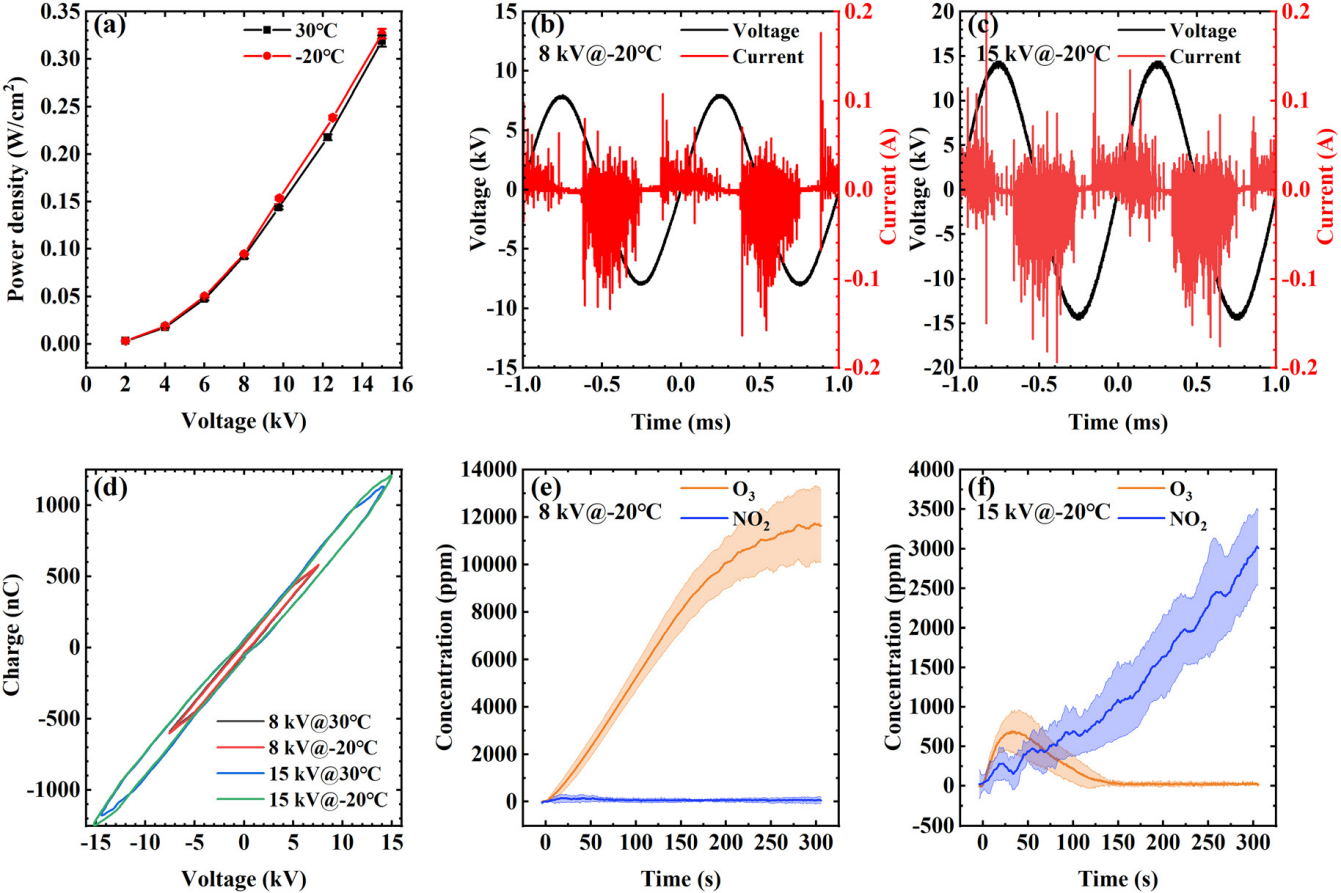
(a) SDBD power density at −20 and 30 °C. (b) and (c) Voltage and current waveforms of 8 and 15 kV at −20 °C. (d) Lissajous figure of 8 and 15 kV at −20 and 30 °C. (e) and (f) Time evolution of a gas concentration of 8 and 15 kV at −20 °C.

The voltage and current waveforms of 8 and 15 kV at −20 °C are shown in Figs. 2(b) and 2(c). The current is composed of a series of small pulses in the frozen environment, showing the characteristics of filamentary discharge, which is consistent with the discharge characteristics of SDBD at room temperature.^23^ There are two typical modes of SDBD at room temperature, namely, the O_3_ mode and the NO_x_ mode, which has been found by other researchers.^27,28^ Shimizu *et al.*^28^ showed that the mode transition was related to the power density, and the threshold was about 0.1 W/cm^2^. Based on the results that the power density is almost the same at the same voltage at both temperatures, it is speculated that these two modes may also exist in the process of SDBD in the cryogenic environment.

UV spectroscopy is performed as an *in situ* measurement of the gaseous composition generated by SDBD at −20 °C. The schematic is shown in Fig. 1. A Teflon gas cell with a cylindrical cavity (40 mm in diameter and 15 mm in depth) in the center is designed. There is a hole (6 mm in diameter) with quartz glass on both sides at 5 mm below the ground electrode. Two optical fibers are connected to a mercury lamp (UV light source) and a spectrometer (HR2000+, Ocean optics), respectively. 253.74 and 435.89 nm are selected as the characteristic wavelengths of ozone and nitrogen dioxide, respectively. (Absorption cross sections are estimated to be 1.15 × 10^−21^ and 5 × 10^−23^ m^2^, respectively.)^29,30^ The spectral intensity of these two wavelengths during the discharge process is recorded, and then the gas concentration can be calculated by Beer's law.

The concentrations of O_3_ and NO_2_ at −20 °C are measured within the first 5 min of discharge when the voltage is 8 and 15 kV, and the results are shown in Figs. 2(e) and 2(f). The evolution process of gas composition and concentration under these two voltages is obviously different. Specifically, when the voltage is 8 kV, the concentration of ozone continues to rise to more than 11 000 ppm within 300 s of discharge, while the concentration of nitrogen dioxide is less than 200 ppm in the whole process. In contrast, when the voltage is 15 kV, the concentration of ozone increases to about 750 ppm in 30 s, then gradually decreases to 0 at 150 s, while the concentration of nitrogen dioxide increases continuously especially after the reduction of ozone, and the maximum concentration can reach 3000 ppm. These results directly prove that there are the O_3_ mode and the NO_x_ mode in SDBD at cryogenic temperature. When the voltage is 8 and 15 kV, the power density is about 0.09 and 0.33 W/cm^2^, respectively, which is consistent with the power density threshold of mode transition proposed by Shimizu *et al.*^28^ They believe that the ozone depletion at high power density is caused by the quenching reactions of nitrogen oxides produced by the reaction of vibrationally excited nitrogen molecules with O atoms.

SDBD is an effective approach for direct surface disinfection^27^ and for indirect disinfection by plasma activated water.^31^ However, its direct disinfection effect on microorganisms in ice at cryogenic temperature is not clear. Schematic diagram of the plasma disinfection process at cryogenic temperature is shown in Fig. 3(a). First, 1 ml of *E. coli* normal saline solution with a concentration of ∼10^7^ CFU/mL was put into a culture dish and frozen in a freezer for more than 12 h to prepare ice containing *E. coli*. Then, the dish was transferred into the gas cell, and plasma treatment of different time/power was carried out at −20 °C. After plasma treatment, the ice was little melted. It took at least 2 min at 37 °C for the ice to melt completely (up to 60 min is to study the disinfection effect of post-treatment time). Finally, the samples were serially diluted and plated on lysogeny broth (LB) agar followed by incubation overnight at 37 °C. Colonies were counted and converted to logarithmic values for evaluating the disinfection effect. The control group is the same volume of bacteria suspension, which is also frozen and melted without plasma treatment. All tests were performed in triplicate to provide a statistically robust comparison.

**FIG. 3.**
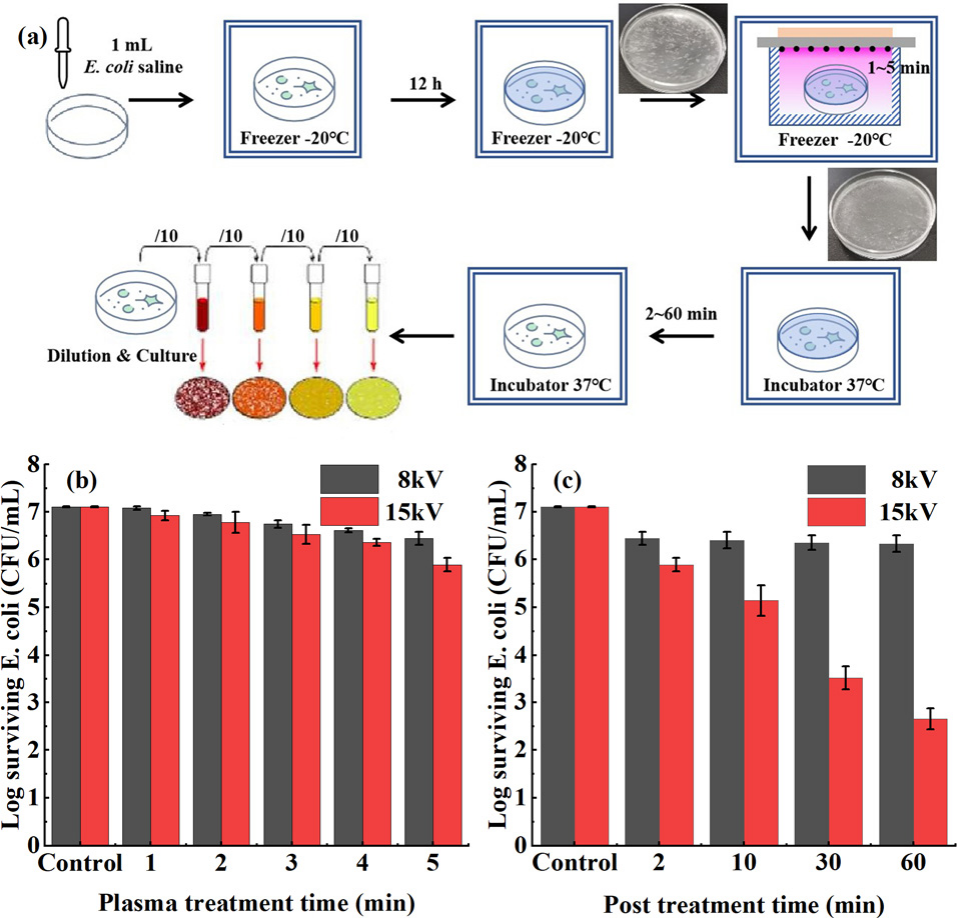
(a) Schematic diagram of the plasma disinfection process at cryogenic temperature. (b) Log surviving *E. coli* number after different plasma treatment time. (The post-treatment time is fixed at 2 min.) (c) Log surviving *E. coli* number after different post-treatment time. (The plasma treatment time is fixed at 5 min.)

The disinfection effect of the O_3_ mode and the NO_x_ mode at cryogenic temperature is studied, including two factors: one is the plasma treatment time (1–5 min), and the other is the post-treatment time (2–60 min). The results are shown in Figs. 3(b) and 3(c). In general, the log of surviving *E. coli* decreased with the treatment time. As shown in Fig. 3(b), the disinfection effect of the two modes is weak under different plasma treatment time. (The post-treatment time is fixed at 2 min.) The log value only decreases by 0.67 and 1.22, respectively, in 5 min when the voltage is 8 kV (O_3_ mode) and 15 kV (NO_x_ mode). However, there is a significant difference in the disinfection effect between the two modes with the increase in post-treatment time. [As shown in Fig. 3(c), the plasma treatment time is fixed at 5 min.] The reduction of log value in the O_3_ mode only increases slightly in 60 min (from 0.67 to 0.78), while it increases significantly to 4.45 in the same time in the NO_x_ mode, showing a good disinfection effect.

Next, the possible disinfection mechanism of these two modes at cryogenic temperature is discussed. Heat and UV are not considered to be the main factors in the process of plasma disinfection,^32^ so the active species in plasmas are mainly considered here. Dozens of reactive oxygen species (ROS) and reactive nitrogen species (RNS) can be produced in the SDBD process, but according to the research results of Liu *et al.*,^33^ most of the active species are short-lived species, and their diffusion distance is less than 1 mm. In this study, ice is placed 15 mm downstream of the SDBD setup, so only long-lived species are considered. As shown in Figs. 2(e) and 2(f), the gaseous products of the two modes are obviously different, one is mainly O_3_ and the other is NO_x_. Considering the relative humidity of 50% in the freezer, there is also the possibility of H_2_O_2_ and HNO_x_. Shimizu *et al.*^28^ and Pavlovich *et al.*^22^ believed that ozone had a good disinfection effect, but the disinfection objects in their study were agar surface or solution. Ozone could directly act on bacteria or diffuse into a solution in their cases. In this study, the whole plasma treatment process is at cryogenic temperature (−20 °C), and the ice is little melted after treatment. Some studies have shown that the uptake coefficient of ozone on the ice surface is very low (<10^−8^).^34^ Therefore, although the ozone concentration produced by SDBD in the O_3_ mode is very high, it will not diffuse into the bulk of ice and may only interact with a small part of *E. coli* exposed on the ice surface, so the disinfection effect is very weak. In the O_3_ mode, H_2_O_2_ may be absorbed on the ice surface (uptake coefficient is about 0.02),^34^ but Aboubakr *et al.*^35^ did not believe that H_2_O_2_ play a key role in their experiments, which explains that the disinfection effect has no obvious change with the increase in post-treatment time in the O_3_ mode. When it comes to the NO_x_ mode, the long-lived gaseous products may include NO_x_, HNO_x_, and H_2_O_2_, all of which are likely to adsorb on or react with ice.^34,36,37^ Then H_2_O_2_ and HNO_2_ can produce peroxynitrite under acidic conditions, which is a powerful disinfection species considered by many researchers.^23,35^ In the 5 min of plasma treatment, as in the case of the O_3_ mode, the disinfection only occurs on the ice surface, so the disinfection effect is also weak. With the increase in post-treatment time, peroxynitrite can diffuse in the solution and inactivate the *E. coli* after ice melting, so it shows a good disinfection effect. The schematic diagram of the above assumed disinfection mechanism is shown in Fig. 4(a).

**FIG. 4.**
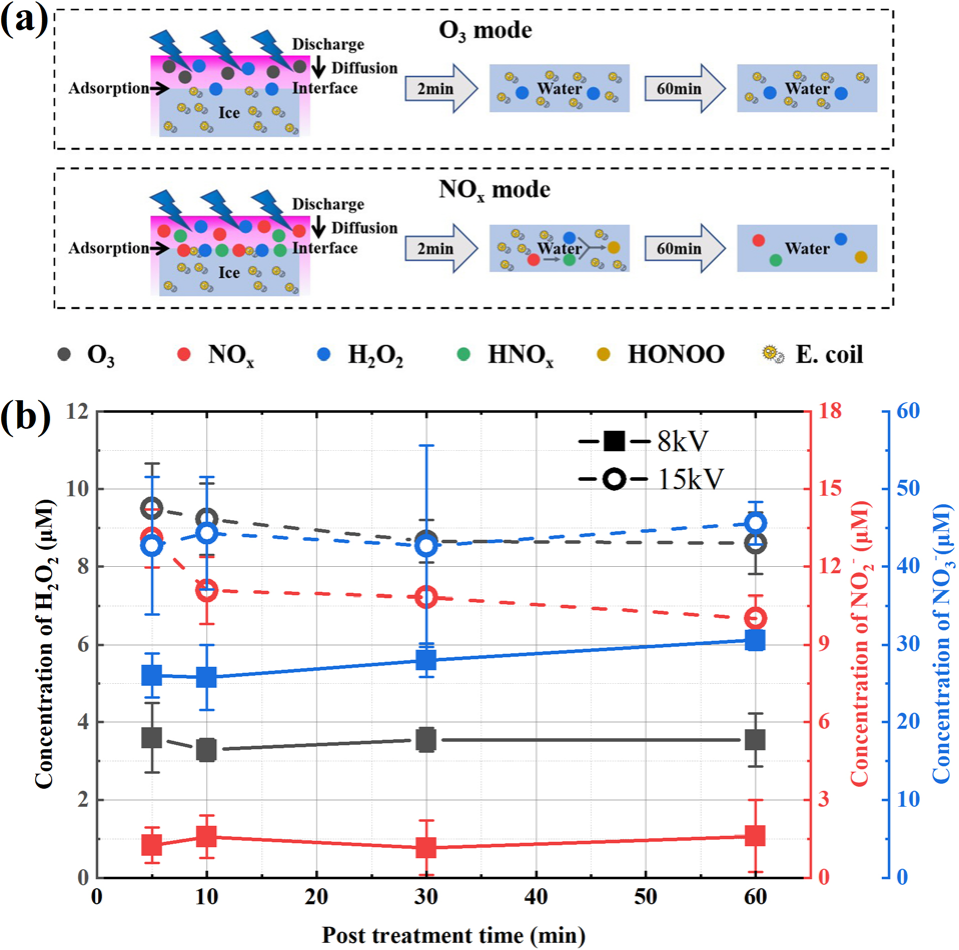
(a) Schematic diagram of assumed disinfection mechanism of the O_3_ mode and the NO_x_ mode. (b) Concentrations of H_2_O_2_, NO_2_^−^, and NO_3_^−^ in the solution after melting ice treated by SDBD for 5 min at 8 kV/15 kV.

In order to verify the above hypothesis, concentrations of H_2_O_2_, NO_2_^−^, and NO_3_^−^ in the solution after melting ice treated by SDBD were further measured, and the curve of concentration with post-treatment time was obtained, as shown in Fig. 4(b). The detection methods of H_2_O_2_, NO_2_^−^, and NO_3_^−^ were described in Ref. 38 using Amplex Red Reagent (Genecopoeia, Rockville, USA) and Griess reagents (Beijing, Shanghai, China), respectively. The results show that the concentrations of the substances in the solution are significantly different in the two modes. In the O_3_ mode, the concentrations of H_2_O_2_ and NO_2_^−^ are about 3.5 and 1.5 *μ*M, respectively, and almost keep constant within 60 min post-treatment time. The concentration of NO_3_^−^ slightly increases from 25 to 30 *μ*M. In the NO_x_ mode, concentrations of H_2_O_2_ decrease from 9.5 to 8.5 *μ*M, the concentration of NO_2_^−^ decreases from 13.5 to 10 *μ*M, and the concentration of NO_3_^−^ slowly increases from 42 to 46 *μ*M. According to the third-order rate constant *k* = 1.1 × 10^3^ M^−2^ s^−1^ for the reaction NO_2_^−^ + H_2_O_2_ + H^+^→ONOOH + H_2_O,^39^ 254 nM peroxynitrite can be accumulated in 60 min post-treatment time in the NO_x_ mode but only 10 nM in the O_3_ mode (*p*H = 3.3). The 25-fold difference of peroxynitrite may be the main reason for the significant difference in the disinfection effect between these two modes.

In conclusion, a low temperature plasma disinfection technique for inactivating *E. coli* in ice at cryogenic temperature was developed. The discharge characteristics of SDBD at cryogenic temperature (−20 °C) are almost the same as those at room temperature, and the power density at the same voltage is similar. Therefore, the mode transition of SDBD determined by power density is also found at −20 °C, and it is the O_3_ mode at 0.09 W/cm^2^ and the NO_x_ mode at 0.33 W/cm^2^. The disinfection effect of these two modes with different plasma treatment time and post-treatment time is investigated. It is found that the disinfection effect is not obvious within 5 min of plasma treatment in both modes, but it increases significantly with the post-treatment time in the NO_x_ mode with 4.45 log reduction. It is speculated that H_2_O_2_ and NO_x_ can be adsorbed on the ice surface in the NO_x_ mode and then converted into a powerful disinfection species, peroxynitrite, showing a good disinfection effect. The concentrations of H_2_O_2_ and NO_2_^−^ in the solution are detected to verify the above hypothesis. Low-temperature plasma is a promising technique for cold chain disinfection, which is of great significance for the protection of people's health and safety. The key disinfection factors and the disinfection mechanism in the cryogenic environment need to be further studied. In addition, a large-scale plasma disinfection device for practical applications needs to be further developed.

## Data Availability

The data that support the findings of this study are available from the corresponding authors upon reasonable request.
